# Twelve tips for teaching medical students about female genital mutilation (FGM)

**DOI:** 10.15694/mep.2019.000117.1

**Published:** 2019-05-30

**Authors:** Jonathan Mayhew, Faye Gishen, Jayne Kavanagh

**Affiliations:** 1 UCL Medical School

**Keywords:** FGM, Female genital mutilation, Female circumcision, Undergraduate medical education, Teaching medical students sensitive topics, Violence against women and girls, Decolonising the curriculum

## Abstract

This article was migrated. The article was marked as recommended.

**Background:** Seeing women or girls from FGM practising communities can be a challenge for healthcare professionals, involving a complex interplay of professional duties, legal requirements, social and cultural understanding, and sensitive, skilled communication.

**Aims and methods:** Robust training on how to identify and support women and girlswho have undergone or who are at risk of FGM **,** including fulfilling FGM-related legal duties,is essential for healthcare professionals. We believe it is important that this training begins in medical school so that junior doctors feel prepared to talk to women and girls from FGM practising communities as soon as they qualify and start work.

**Results:** We have reviewed the limited existing literature on teaching medical and other healthcare students about FGM and have drawn on our many years of providing well-evaluated teaching sessions on FGM at UCL Medical School to design twelve tips as a blueprint for running sensitive and effective undergraduate teaching on FGM.

**Conclusions:** Effective teaching for medical students on FGM is important and feasible **.** Curricula leads and individual teachers will benefit from a structured, comprehensive and culturally sensitive approach outlined in the twelve tips.

## Introduction

We use the term female genital mutilation (FGM) as it is widely used and understood in clinical practice and in legal and policy documents. However, we acknowledge the term is justifiably contested and can have a detrimental effect on those who have undergone the practice. We therefore advocate using the term ‘cut’, rather than ‘mutilation’ in discussions with patients from FGM-practicing communities.

FGM comprises all procedures that involve the removal of, or injury to, any part of the female genitals for non-medical reasons (WHO, 2008). Although it is difficult to estimate the number of women in England and Wales who have undergone FGM ( Macfarlane, 2019), NHS England states that every local authority in England is likely to include women who have experienced FGM ( NHS England, 2018). It is therefore essential that healthcare professionals (HCPs) receive effective training on supporting women who have undergone FGM, identifying girls who are at risk of FGM and on fulfilling their FGM-related legal duties.

In 2003 the Royal College of Obstetricians and Gynaecologists was one of the first UK medical bodies to issue formal guidance on this ( RCOG, 2015). However, a systematic review of healthcare professionals’ knowledge about FGM demonstrated that levels of understanding were highly variable ( Zurynski *et al*., 2015). The review highlighted that even professionals working regularly with women from communities where FGM is common felt they had inadequate training about identifying and supporting these women and girls. Furthermore, evidence suggests HCPs often find approaching FGM in practice in a culturally sensitive way difficult ( Hussain & Rymer, 2017). A national e-learning package made accessible to all healthcare professionals may have gone some way to addressing this lack of knowledge, skills, and confidence ( NHS, 2015). Incorporating effective training into undergraduate curricula would further address this and equip newly qualified HCPs with the knowledge and skills they need to support women and girls from FGM-practising communities.

The twelve tips draw on both the limited research on how this topic is addressed in undergraduate healthcare professional education, and on our experience of delivering formal, well-evaluated FGM teaching sessions for several years at our large UK medical school.

## Tips

### Tip 1 Develop understanding of the cultural and social complexity of FGM before the teaching session

FGM is a deeply embedded social norm, practised by communities and families for a variety of complex reasons and is often thought to be essential for a girl to become a proper woman and to be marriageable. In order to engage students in the topic and give them insight into this complexity, we ask them to watch a documentary commissioned by the International Federation of Gynecology and Obstetrics (The Cutting Tradition 2012) before the formal teaching session.

### Tip 2 Acknowledge sensitivity of the topic and provide staff contact for debriefing and support

FGM can be a sensitive and upsetting topic. Students in the teaching session may know someone who has experienced FGM (a friend, relative, patient, NHS staff member), be concerned that they know someone is at risk of FGM or may have undergone FGM themselves. It is important to acknowledge this at the start of the teaching session and inform students that they can leave at any time. It is also worth reminding students that they should be mindful of this when discussing and asking questions about FGM in the session. Giving students warning of potentially sensitive content in advance allows them to deploy strategies for dealing with any difficult memories that may surface during the session ( Collins, 2013).

Furthermore, Kennedy & Scriver (2016) stress the importance of providing a person to contact should students wish to discuss their emotional response to being taught about a sensitive and potentially upsetting topic. In our session, the facilitator makes themselves available to speak to students directly afterwards and provides contact information for those wishing to access further support.

We recommend all facilitators read the “Do No Harm” guidance produced by Girl Generation (2014) to help them gain understanding about how to avoid stigmatising or causing emotional distress to those who have undergone FGM: for example exercising caution around the use of graphic images of FGM.

### Tip 3 Bring basic factual knowledge to the same level

Learners may come from very different cultural backgrounds and may have different educational and clinical experience, predisposing them to different levels of knowledge about FGM. Use of a quiz or similar learning tool can both assess and bring learners to the same baseline level of knowledge in an interactive and engaging way. A quiz can be completed individually or in pairs using a physical handout to stimulate peer discussion or via an online voting platform.

The quiz (see Appendix) ensures a group of diverse learners, who may practise in many different healthcare settings in the future, understand the fundamentals of FGM without overburdening them with specialist knowledge. Concerningly, a study of 45 healthcare professionals in a teaching hospital in London found only 4% could correctly identify the four types of FGM ( Zaidi *et al*., 2007). The quiz addresses gaps in knowledge like this by including questions on basic information such as the WHO classification system (types 1-4). When going through the answers to the quiz the facilitator can evoke discussion on current law, prevalence, process, and complications of FGM.

### Tip 4 Ensure understanding of potential complications of FGM

The consequences of FGM are variable and wide-ranging, including changes to women’s roles in their community and social standing, as well as effects on physical and mental health. It is essential doctors are aware of these potential physical and psychological consequences of FGM in order to provide appropriate treatment and support, especially as many women with FGM may not actively seek help for problems related to FGM.

There is currently nothing in the published literature regarding UK medical students’ knowledge about FGM. However, a study that assessed medical students’ knowledge about FGM in Egypt ( Mostafa *et al*., 2006) found that less than half of the cohort of Egyptian medical students surveyed were aware FGM could lead to physical complications, even in the context of a high prevalence of FGM in Egypt.

Despite there being no data on medical students’ knowledge in the UK, there is evidence that qualified doctors in the UK have variable levels of knowledge. A study of obstetricians and gynaecologists showed that even though 92% of respondents were able to identify the long term physical complications of FGM, only 9% identified psychological complications ( Purchase *et al*., 2013). Sex, pregnancy, and childbirth pose further challenges to women who have experienced FGM, and it is crucial to include these topics as part of core learning about FGM.

In our teaching session, the quiz ensures early understanding of the potential complications of FGM (Appendix, questions 7 and 8).

### Tip 5 Critically evaluate justifications and explain facts

One of the biggest challenges to combating FGM worldwide is exposing and challenging misconceptions associated with the practice. Providing students with an understanding of the context and beliefs surrounding FGM and the justifications given for performing it not only enhances their understanding of the complex cultural processes involved but ensures they are adequately informed and prepared to refute justifications and explain facts to future patients from FGM-practising communities.

Common misconceptions are addressed in the quiz questions (Appendix, questions 10 and 11), and the reasons families allow their daughters to undergo FGM also discussed. These include justifications such as protecting chastity and fertility, improving marriageability and community belonging, and mistaken beliefs such as the clitoris will grow to the size of a penis if not removed and the baby will die if it touches the clitoris during childbirth. The common belief that FGM is a religious requirement is presented in a sensitive manner using quotations from prominent religious figures who have condemned FGM.

It can be challenging for some students to understand how parents could allow their daughters to undergo FGM and it is important the session addresses this so that students understand that unlike most forms of child abuse, loving parents can genuinely believe that having FGM is in their daughters’ best interests, and is essential for community belonging and marriageability.

### Tip 6 Clarify current law and legal requirements

The law relevant to FGM in the UK is evolving. As with all medico-legal teaching, it is essential that updates to legislation are reflected in contemporary teaching materials so that students have a clear understanding of current law and their legal duties. Our session outlines the requirements of the Female Genital Mutilation Act 1985, as amended in 2003, and the Serious Crime Act 2015. Healthcare professionals’ legal duty to report all under 18s who disclose or have evidence of FGM to the police is emphasised, as is the requirement for health professionals to submit patient data to the FGM enhanced dataset. A discussion of the Whittington Health NHS Trust legal case, where a doctor was charged with performing FGM but later acquitted ( Dyer, 2015), facilitates an appreciation of the complexities surrounding the legality of FGM relevant to clinical practice and stimulates some critical thinking about the law related to FGM (see Tip 10).

### Tip 7 Develop skills to identify and support women who have undergone FGM

It is essential that doctors have both the knowledge and communication skills to identify women from FGM-practising communities so that they can effectively treat current medical issues and offer/signpost to support. Our teaching concentrates on this, firstly acknowledging that evidence shows that healthcare professionals often find it difficult to know how to approach FGM in a culturally sensitive manner ( Hussain & Rymer, 2017) and thinking about why this might be. Students participate in a role-play where they sensitively ask whether the person sat next to them has undergone FGM. Respectfully raising the topic of FGM in consultations is discussed, using verbatim phrases such as, ‘Have you ever had any operations on your genitals or genital piercings or had FGM, or cutting or circumcision?’ Questions that identify physical and psychological consequences and support needs are discussed and examples given, including questions about flashbacks and nightmares.

In order to reinforce learning points and increase their confidence to ask about FGM, students watch a short video of a medical student speaking to a woman from Sierra Leone in a general practice surgery who has symptoms of a urinary tract infection. The video demonstrates how, even as students, HCPs can sensitively and effectively ask a woman about FGM and help support her. The video is freely available online as a learning resource ( UCL Medical School, 2017).

Drawing students’ attention to relevant clinical guidance, for example on procedures for de-infibulation and re-stitching after childbirth and on post-maternity conversations on safeguarding ( RCOG, 2015; RCN, 2019) will help them learn how to support women with FGM in maternity care and identify girls are risk of FGM (see Tip 8).

### Tip 8 Develop skills to indentify and support girls at risk of FGM

As well as identifying and supporting women who have experienced FGM, learners must also be equipped to identify girls at risk of FGM. We describe useful questions for risk assessment to use in consultations and outline good safeguarding practice when clinicians identify an immediate risk of FGM. We also discuss what clinicians can say to people from FGM-practising communities to help prevent future FGM. The video mentioned in Tip 7 reinforces these learning points.

### Tip 9 Involve women with experience of FGM

The opportunity to hear from a woman who has personally undergone FGM is an incredibly valuable learning experience for students. Existing literature suggests that narratives tap into several key learning processes in medical education, including providing a relevant context for understanding, engaging learners, and promoting memory and empathy ( Easton, 2016).

Hearing a first-hand account of the cultural significance of FGM in a specific community and the detrimental impacts of FGM on an individual’s health and wellbeing, as well as personal experience of encounters with healthcare professionals reinforce the importance of everything students have learned in the session. Our speaker, SKK, discusses her experiences freely with students and encourages them to ask any questions, helping break down taboos and concerns about discussing FGM with patients. The passion with which she speaks about ending the practice and how doctors can help with this, as well as how to support women who have undergone FGM promotes understanding and empathy in the students and inspires them to be competent and caring clinicians with a desire to help tackle the issue. As one student said, “Really brilliant teaching, particularly the opportunity to hear from [a] survivor of FGM”. Another student reports, “I’ll never forget it”; and another, says “Hearing women’s personal stories about FGM..was very touching and so helpful for us to understand why these things happen and what we can do to help”.

It is important to note thatnot everyone who has undergone FGM is happy to share their experience with students and to ensure that those who are willing to speak out are supported, in order to minimise the risk of being re-traumatised.

### Tip 10 Discuss ethical issues

Ensuring students are familiar with legal and professional guidance on FGM is integral to the session. However, aspects of current law and certain statutory duties regarding FGM such as mandatory reporting ( Creighton *et al*., 2019) and data sharing ( Kelly, 2016) are ethically controversial. Our session gives students the opportunity to critically appraise FGM legislation and policy and discuss this in relation to legal duties, confidentiality, best interests and unintended consequences of legal requirements.

These discussions help students understand that sensationalising of the issue and ‘zero tolerance’ approaches that encourage clinicians to consider patients solely through a ‘safeguarding lens’ may demonise women who have experienced FGM and risk unintended consequence of alienating them from accessing physical and psychological care ( Creighton & Bewley, 2018).

Time permitting, it can be enlightening to expand discussion to more political issues such as the motivation for FGM-related prosecutions and how the warning that quantification of the numbers of women and girls in the UK who have experienced FGM is not possible has largely been ignored ( Macfarlane, 2019). Additional discussion around similarities and differences with male infant circumcision and intersex surgery and the increasing medicalisation of FGM in countries including Egypt and Sudan (where some doctors perpetuate the practice), can be both challenging and instructive.

### Tip 11 Use expert facilitators

The ideal facilitator for an effective and comprehensive teaching session on FGM for medical students is a clinician with experience of caring for women/girls from FGM practising communities who also has experience of teaching healthcare professionals/students on sensitive topics, including medical ethics and law. However, it may be difficult to find all these skills and experience in one person. Identifying a number of facilitators with some of the above skills and qualities and further training them to deliver these sessions, in combination with co-facilitation and constructive peer-review of teaching sessions, can provide good training and support to facilitate effective learning.

### Tip 12 Highlight students’ potential to be an advocate for change

At the end of our session, we remind students that as future medical professionals, often held in high esteem by local communities, they could play a pivotal role in education and approaches to FGM, both at a local level with individual patients, but also at a wider political level. By harnessing their medical expertise to demystify myths around FGM, and working with other agencies, academics, the government and policy makers they have potential to become key proponents for promoting change and ending this harmful practice.

## Conclusion

These twelve tips are based on our many years of experience of teaching medical students about FGM and on best available evidence. We hope that by sharing our practice we can help medical educators, as well as those teaching other healthcare learners in undergraduate and postgraduate settings, to deliver effective teaching sessions on FGM. We hope this will help more medical students feel comfortable about speaking to women about FGM, and more doctors become competent at recognising, treating and supporting women who have undergone FGM as well as identifying girls who are at risk.

## Take Home Messages

Effective teaching on FGM:

• Requires careful planning and skilled facilitation
• Is culturally and emotionally sensitive
• Incorporates relevant law, ethical issues and communication skills
• Equips learners with the knowledge and skills they need to identify and support women and girlswho have undergone FGM or who are at risk of FGM

## Notes On Contributors

Dr Jonathan Mayhew is an Honorary Clinical Teaching Fellow at UCL Medical School, London, UK.

Dr Faye Gishen is a consultant physician and academic lead for Clinical and Professional Practice at UCL Medical School, London, UK.

Dr Jayne Kavanagh is a Principal Clinical Teaching Fellow at UCL Medical School and Specialty Doctor in Sexual Health at Homerton University Hospital NHS Foundation Trust.

## Appendices

Quiz on knowledge and understanding of FGM

1. List at least two terms by which FGM is also known.

2. How many women and girls worldwide are estimated to have undergone FGM?

a) 20 - 30 million b) 50 - 75 million c) 100 - 140 million

3. Underline the countries where over 70% of women are estimated to have undergone FGM.

Pakistan Egypt

Sudan Mali

India Indonesia

Tanzania Burkina Faso

Nigeria Chad

Iraq Ethiopia

Oman Yemen

Eritrea Guinea

Mauritania Somalia

Togo Senegal

Kenya Sierra Leone

4. What is the most extreme form of FGM?

a) Type 1 b) Type 2 c) Type 3 d) Type 4

5. The most extreme form of FGM constitutes what % of FGM worldwide?

a) 15% b) 30% c) 65%

6. True or false? In Egypt 70% of FGM is performed by healthcare practitioners.

a) True b) False

7. Which of the following is not a potential short-term complication of FGM?

a) Shock b) Bleeding c) Infection d) DVT

e) Urine retention f) Severe pain g) Death

8. Which of the following is not a potential long-term complication of FGM?

a) Chronic pain

b) Chronic infections

c) Hernia

d) Difficulties with menstruation and passing urine

e) Sexual problems

f) Difficulty conceiving/infertility

g) Mental health and psychological problems (PTSD)

h) Obstetric complications

9. True or False? It is legal for a UK citizen or resident to arrange for her child to undergo FGM provided it is not carried out in the UK.

a) True b) False

10. Which of the following is not a reason given for performing FGM?

a) Custom and tradition

b) Social acceptance, especially for marriage

c) Family honour and sense of belonging to the group

d) Fear of social exclusion

e) Preservation of virginity/chastity

f) Increased chance of giving birth to a boy

g) Religion

h) Increasing sexual pleasure for the male

i) Enhancing fertility

j) Hygiene and cleanliness

11. Which of the following is not a myth about FGM?

a) The clitoris can cause male impotence

b) If the clitoris is not removed it will grow to the size of a penis

c) The baby will die if it touches the clitoris during birth

d) The clitoris will start smelling at puberty

e) The clitoris represents the male soul and needs to be removed in order for adolescents to enter adulthood

12. Which of the following symptoms in a child could be caused by recent FGM?

a) Difficulty walking

b) Difficulty sitting

c) Difficulty standing

d) Long time spent in bathroom/toilet

e) Prolonged absence from school

f) Behaviour changes e.g. depression

13. Imagine you are an FY doctor working in the Emergency Department. You see a 32-year woman born in Sierra Leone with lower abdominal pain and irregular vaginal bleeding. Would you ask her a question about FGM?

a) Yes b) No

## Declarations

The author has declared that there are no conflicts of interest.

## Ethics Statement

This is a 12 tips paper. Ethical approval was not required.

## External Funding

This article has not had any External Funding

## References

[ref1] Collins A. (2013) Teaching sensitive topics: Transformative pedagogy in a violent society. International Special Edition. 9, pp.28–149.

[ref2] Creighton S. and Bewley S. (2018) Re: Tackling female genital mutilation in the UK. The Obstetrician and Gynaecologist. 20(3), pp.209–210. 10.1111/tog.12502

[ref3] Creighton S.M. Samuel Z. Otoo-Oyortey N. and Hodes D. (2019) Tackling female genital mutilation in the UK. BMJ. 364(15). 10.1136/bmj.l15 30617106

[ref4] Dyer C. (2015) More doctors should be trained in identifying female genital mutilation in children, says judge. BMJ. 350(273). https://www.bmj.com/content/350/bmj.h273 10.1136/bmj.h273 25601462

[ref5] Easton G. (2016) How medical teachers use narratives in lectures: a qualitative study. BMC Medical Education. 16(3). 10.1186/s12909-015-0498-8 PMC4705637 26742778

[ref6] Girl Generation , (2014) Do No Harm. Available at: https://www.thegirlgeneration.org/sites/default/files/files/FINAL%20The%20Girl%20Generation%20Do%20No%20Harm%20Guidelines.pdf( Accessed: 22 February 2019)

[ref7] Hussain S. and Rymer J. (2017) Tackling female genital mutilation in the UK. Obstetrics and Gynaecology. 19(4), pp.273–278. 10.1111/tog.12394

[ref8] Kelly B. (2016) We need better data on FGM, not propaganda, The Guardian. Available at: https://www.theguardian.com/society/2016/jul/31/we-need-better-data-on-fgm-not-propaganda( Accessed: 17 June 2018)

[ref9] Kennedy K. M. and Scriver S. (2016) Recommendations for teaching upon sensitive topics in forensic and legal medicine in the context of medical education pedagogy. Journal of Forensic and Legal Medicine. 44, pp.192–195. 10.1016/j.jflm.2016.10.021 27835836

[ref10] Macfarlane A. J. (2019) Misleading use of FGM statistics compounds concerns about their reliability. BMJ. 364(1927). 10.1136/bmj.l927 30837267

[ref11] Mostafa S.R.A. El Zeiny N. a. M. Tayel S.E.S. Moubarak E.I. (2006) What do medical students in Alexandria know about female genital mutilation? Eastern Mediterranean Health Journal. 12(2), pp.78–92. 17361680

[ref12] NHS (2015) Female Genital Mutilation, E-Learn Health. Available at: https://www.e-lfh.org.uk/programmes/female-genital-mutilation/( Accessed: 19 October 2017)

[ref13] NHS England (2018) Commissioning services to meet the needs of women and girls with FGM. Available at: https://www.england.nhs.uk/wp-content/uploads/2018/04/commissioning-services-to-meet-the-needs-of-women-and-girls-with-fgm-1.pdf( Accessed: 19 February 2019)

[ref14] Purchase T.C.D. Lamoudi M. Colman S. Allen S. (2013) A survey on knowledge of female genital mutilation guidelines. ACTA Obstetricia et Gynecologica Scandinavica. 92, pp.858–861. 10.1111/aogs.12144 23581719

[ref15] RCN (2019) Safeguarding Children and Young People: Roles and Competencies for Healthcare Staff. Available at: https://www.rcn.org.uk/professional-development/publications/007-366( Accessed: 19 February 2019)

[ref16] Royal College of Obstetricians and Gynaecologists (2015) Female Genital Mutilation and its Management. Available at: https://www.rcog.org.uk/globalassets/documents/guidelines/gtg-53-fgm.pdf( Accessed: 19 February 2019)

[ref17] Safe Hands for Mothers (2012) The Cutting Tradition: Insights into female genital mutilation. Available at: https://www.youtube.com/watch?v=pUpToERm0q0( Accessed: 22 December 2017)

[ref18] UCL Medical School (2017) FGM Student Consultation. Available at: https://mediacentral.ucl.ac.uk/Player/70484128( Accessed: 17 June 2018)

[ref19] World Health Organisation (2008) Eliminating female genital mutilation. Available at: https://www.who.int/reproductivehealth/publications/fgm/9789241596442/en/( Accessed: 21 February 2019)

[ref20] Zaidi N. Khalil A. Roberts C. and Browne M. (2007) Knowledge of female genital mutilation among healthcare professionals. Journal of Obstetrics and Gynaecology. 27(2), pp.161–164. 10.1080/01443610601124257 17454465

[ref21] Zurynski Y. Sureshkumar P. Phu A. and Elliott E. (2015) Female genital mutilation and cutting: a systematic literature review of health professionals’ knowledge, attitudes and clinical practice. BMC International Health and Human Rights. 15(32). 10.1186/s12914-015-0070-y PMC4676087 26652275

