# Supplementary material for: Twelve tips for teaching medical students about female genital mutilation (FGM)
Source: MedEdPublish (2016). 2019 May 30;8:117. [Version 1] doi: 10.15694/mep.2019.000117.1 (PMC10712467; doi:10.15694/mep.2019.000117.1)
Supplement: Peer review history

# Reviewer report: Reviewer response for version 1

**DOI:** 10.21956/mep.19841.r29920

**Published:** 2019-07-12

**Authors:** Trevor Gibbs

**Affiliations:** 1 AMEE

This review has been migrated. The reviewer awarded 5 stars out of 5 An excellent paper, about a difficult but very important subject- well doneI learned much from this paper and I was very pleased to see that the authors were prepared to base the paper on their experiences and not just look for researched evidence- experience counts for much in this field. Like my co-reviewer I would have liked the answer key to the questions, but then I think this demonstrates the value of this paper by leaving the reader wanting to know more.A key paper to be read by anyone in the health professions

Reviewer Expertise:

NA

No decision status is available

# Reviewer report: Reviewer response for version 1

**DOI:** 10.21956/mep.19841.r29921

**Published:** 2019-06-28

**Authors:** Alexander Woywodt

**Affiliations:** 1 Lancashire Teaching Hospitals NHS Foundation Trust

This review has been migrated. The reviewer awarded 5 stars out of 5 Mayhew and colleagues take up the important topic of female genital mutilation (FGM) and write for a broad audience of educators, faculty and policy makers. It is somewhat surprising that so far this topic, which has received widespread attention in the medical community as well as coverage in lay media, has not been addressed by medical educators in any great detail. A PubMed search on “female genital mutilation” and “education” as title words only retrieves 30 or so titles, most of whom deal with educating communities or the public. Mayhew and colleagues make a valid point here i.e. that awareness of FGM could be much improved by including this topic into undergraduate education. I find their paper very readable and their twelve tips well thought through. I think the caveats they emphasise are also well taken for example the notion that seeing FGM solely through the lens of safeguarding may not do the affected women a great service and may lead to women avoiding contact with health care practitioners. At times one would like to see the authors dig a little deeper for example when they allude to the political context of FGM – one or two additional references on this aspect would have been nice. Perhaps a case vignette involving undergraduates could help to get the message across and illustrate the issues. I appreciate that this is a very sensitive topic but the authors mention their work with an affected woman and I wondered whether an anonymised case vignette could have been presented. I also liked the idea of the quiz but wasn’t quite sure where to find the correct answers. Reading this I also wondered whether the picture described by these authors from London also applies elsewhere in the UK and whether there are regional variations in the prevalence of FGM. Similarly I wondered whether there is variation between the UK and other countries. Perhaps an online survey of medical educators could be interesting to get an idea of awareness and current practice i.e. whether FGM is part of the curriculum or addressed in any formal or informal way. I have also shared this paper with my colleagues in Year 4 teaching where gynaecology sits in our curriculum. Overall I found this a really good paper and would recommend this to a wide audience – well deserving five stars.

Reviewer Expertise:

NA

No decision status is available

# Reviewer report: Reviewer response for version 1

**DOI:** 10.21956/mep.19841.r29922

**Published:** 2019-06-23

**Authors:** Ken Masters

**Affiliations:** 1 Sultan Qaboos University

This review has been migrated. The reviewer awarded 5 stars out of 5 An interesting and well-written paper on an extremely difficult, delicate and profoundly disturbing subject. My opening thoughts are to congratulate the authors on writing this much-needed paper. The authors have presented their material carefully and as straight-forwardly as possible, and in a way that clearly identifies the issues and yet does not become entangled in distracting information. I also particularly like the fact that the authors have supplied a useful mixture of academic and non-academic resources for faculty to use in teaching the topic.Given the lack of available literature, the authors have had to draw heavily on their own experience. Of necessity, then, the paper’s geographical role is focused on the UK, and it is fair to expect the authors to confine themselves to that area, as one could imagine that every country will have different aspects of law and cultural practices to take into account.In spite of the localisation, the paper has important lessons for medical educators and medical schools across the world, and provides a solid basis upon which others may build; I would also like to see other researchers and practitioners take up the topic and extend the work into their own area, so that a global picture can be created.(It is a pity that the authors have not supplied an answer key to their quiz – although that may serve as a useful self-study exercise for medical educators. On the other hand, if the authors are prepared to share their answer key (or at least, “grade” an answer from interested medical educators), it might serve to assist others in their path to teaching the topic. Perhaps the authors may consider specifically inviting other medical educators to contact them on this issue).A fine paper, well-deserving of 5 stars.

Reviewer Expertise:

NA

No decision status is available
